# The Hippocampus Links Episodic Memory and Bodily Awareness

**DOI:** 10.1002/brb3.71570

**Published:** 2026-07-08

**Authors:** Lucie Bréchet, Konstantin Toussas, Damien Marie, Paul G. Unschuld

**Affiliations:** ^1^ Department of Clinical Neurosciences University of Geneva Geneva Switzerland; ^2^ Center For Biomedical Imaging, Cognitive and Affective Neuroimaging Section University of Geneva Geneva Switzerland; ^3^ Geriatric Psychiatry Service University Hospitals of Geneva (HUG) Thônex Switzerland; ^4^ Department of Psychiatry University of Geneva Geneva Switzerland

**Keywords:** autonoetic consciousness, bodily self‐consciousness, episodic memory, hippocampus, mild cognitive impairment

## Abstract

**Purpose:**

Episodic autobiographical memory does not occur in isolation from the body: every remembered event was once encoded from a first‐person perspective. Yet whether the hippocampus, long recognized as the canonical substrate of memory, also underpins the embodied self has remained unknown.

**Method:**

We combined structural T1‐weighted MRI with behavioral assessments of episodic memory (Logical Memory delayed recall) and bodily awareness (ARSQ 2.0 Somatic Awareness subscale) in patients with mild cognitive impairment (MCI) and healthy comparison groups to test whether hippocampal volume predicts both outcomes.

**Finding:**

We show that hippocampal volume predicts not only episodic memory performance but also bodily awareness in patients with MCI. Hippocampal volume explained substantial variance in both domains, whereas a control region did not, underscoring regional specificity. This effect was paralleled by a reduction of hippocampal volume in MCI patients compared to older and younger adults. Notably, both high‐ and low‐cognition older adults showed larger volumes than in MCI.

**Conclusion:**

These findings provide the first structural evidence that hippocampal atrophy is associated with reductions in both remembering and embodiment, positioning the hippocampus as a key node linking memory and bodily selfhood. Beyond revealing a novel dimension of hippocampal vulnerability, this work suggests translational opportunities. These findings open the possibility that bodily awareness may prove useful as an early marker in future longitudinal studies of Alzheimer's disease.

## Graphical abstract

Hippocampal volume predicts both memory and bodily awareness in patients with mild cognitive impairment. This structural link positions the hippocampus as a key node connecting memory and the embodied self, with implications for early detection of Alzheimer's disease.

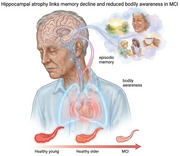

## 1 Introduction

Episodic memory is inherently embodied: every remembered event was once encoded from within one's own body. Bodily self‐consciousness (BSC), the multisensory integration of signals that grounds the sense of being a self in a body (Blanke 2012), critically shapes how memories are formed and recalled. Experimental manipulations show that encoding events from a first‐person bodily perspective enhances later recall compared to disembodied or third‐person perspectives (Bréchet et al. 2020; Iriye and Ehrsson 2022; Bréchet et al. 2019; Bergouignan et al. 2014). These findings suggest that the bodily self constitutes a core dimension of episodic remembering rather than merely providing contextual cues. Beyond multisensory exteroceptive cues, ongoing cardiac and respiratory interoceptive rhythms shape early awareness‐related brain activity, indicating that bodily signals can gate access to conscious perception (Leupin and Britz 2024).

Converging evidence suggests that the hippocampus serves as a hub for this body‐memory interaction. Hippocampal activity and connectivity track the degree of embodiment (used here broadly to denote the role of the body in shaping memory and self‐experience; see also Bréchet 2022) during encoding and retrieval (Iriye et al. 2024; Gauthier et al. 2020; Meyer et al. 2024). The hippocampal‐parietal network has been implicated in both BSC and episodic autobiographical memory (Bréchet et al. 2018; Iriye and St Jacques 2025).

Crucially, recent clinical evidence provides causal support for this finding. A single‐case study (Meyer et al. 2025) described an amnesic patient with bilateral hippocampal atrophy who exhibited profound deficits in episodic autobiographical memory specifically tied to impaired embodiment at encoding. Strikingly, the usual memory benefit of first‐person embodiment was reversed: the patient recalled more vividly under disembodied conditions. Connectivity analyses revealed disrupted hippocampal‐parietal interactions, underscoring the necessity of hippocampal integrity for enhancing memory and the bodily self.

Together, these findings highlight the hippocampus as a hub linking BSC with episodic memory, enabling the autonoetic re‐experiencing of past events as one's own. Yet despite converging behavioral and neuroimaging evidence, the structural basis of this link remains unknown. Mild cognitive impairment (MCI) offers a unique model: while hippocampal atrophy and memory decline are well‐documented, potential disruptions in bodily awareness (operationalized as self‐reported awareness of interoceptive bodily signals) have not been tested. Here, we combined behavioral testing with structural neuroimaging in MCI and healthy comparison groups. We hypothesized that hippocampal volume would predict both episodic memory and bodily awareness, reflecting a shared structural substrate. By extending prior experimental findings to the prodromal stage of Alzheimer's disease, our study probes whether hippocampal atrophy is associated with alterations not only in memory but also in the embodied self—the experience of the self as grounded in and shaped by the body.

## 2 Methods

We combined behavioral testing with structural neuroimaging to investigate whether hippocampal volume is linked to episodic memory and bodily awareness in MCI. Participants underwent high‐resolution T1‐weighted MRI and behavioral assessments. Structural analyses included hippocampal volume regressions with behavioral measures, group‐level comparisons across age and cognitive status, and whole‐brain voxel‐based morphometry (VBM) analysis.

### 2.1 Participants

A total of 148 individuals were enrolled in this study: 58 healthy young adults (HY) (32 female, mean age = 25.79 ± 4.17 years), 58 healthy older adults (HO) (33 female, mean age = 71.31 ± 8.04 years), and 32 MCI patients (13 females, mean age = 71.53 ± 6.17 years). Of these, 25 had complete behavioral data for both measures and were included in the regression analyses; the remaining seven were excluded because they had missing scores on at least one measure. All participants reported no history of psychiatric or neurological illness other than MCI. Patients were recruited at the Memory Clinic of Geneva University Hospitals and diagnosed by qualified neurologists. Inclusion and exclusion criteria were based on established clinical standards and are described in detail elsewhere (Tarailis et al. 2025). All participants provided written informed consent in accordance with the Declaration of Helsinki, and the study protocol was approved by the Cantonal Ethical Committee of Geneva (CCER; approval number: 2021‐01388).

### 2.2 Behavioral Measures

A comprehensive neuropsychological battery was administered to all MCI patients, as detailed elsewhere (Tarailis et al. 2025). For the present study, two measures were selected from this battery. Episodic memory was assessed by the delayed recall score from the Logical Memory Story B test, which was chosen as a sensitive marker of hippocampal‐dependent long‐term memory. Bodily awareness was assessed with the Amsterdam Resting‐State Questionnaire (ARSQ, version 2.0) (Diaz et al. 2014). The ARSQ 2.0 encompasses 10 dimensions of resting‐state experience—somatic awareness, self, sleepiness, comfort, health concern, discontinuity of mind, theory of mind, planning, visual thought, and verbal thought (see Table S1). In line with our a priori hypothesis focusing on BSC, analyses were limited to the Somatic Awareness subscale, as it is the only dimension directly capturing interoceptive and bodily self‐referential experience. The three items of this subscale are “I was conscious of my body,” “I thought about my heartbeat,” and “I thought about my breathing.” Internal consistency of the three somatic awareness items was *α* = 0.53 (*N* = 31; inter‐item *r* = 0.21–0.40), consistent with a multidimensional instrument sampling distinct facets of interoceptive experience. We note that this level of internal consistency reflects the heterogeneity of the somatic‐awareness construct and may limit direct comparability with more homogeneous psychometric scales. The specificity of this subscale's association with hippocampal volume, which was confirmed against all other ARSQ 2.0 dimensions, nonetheless provides empirical support for its construct validity in the present sample. These measures were then entered into regression analyses with hippocampal volume as the predictor. Global cognitive status was assessed in all participants using the Montreal Cognitive Assessment (MoCA) (Nasreddine et al. 2005). MoCA scores were used to stratify the HO group into high‐ and low‐performing subgroups (cutoff = 26).

### 2.3 MRI Data Acquisition

Structural MRI data were acquired at Campus Biotech (Geneva) using a 3T Siemens Magnetom PRISMA scanner (Siemens Healthineers, Erlangen, Germany). High‐resolution T1‐weighted images were obtained with a 3D magnetization‐prepared rapid gradient‐echo (MPRAGE) sequence using the following parameters: TR = 2300 ms, TE = 2.25 ms, TI = 900 ms, voxel size = 1 × 1 × 1 mm^3^, field of view = 256 mm, slice thickness = 1 mm, 208 slices, and flip angle = 8°.

### 2.4 Associations Between Hippocampal Volume and Behavior

We used multivariate linear regression to test whether hippocampal volume predicted episodic memory and bodily awareness. Hippocampal volume values (derived from the neuromorphometrics atlas [Mangesius et al. 2022]) were first residualized for age and total intracranial volume (TIV) using linear regression. The resulting residuals were used as predictor variables in subsequent analyses. Analyses were conducted in SPSS (Version 29, IBM Corp.) using the general linear model (GLM) procedure, with hippocampal residuals entered as the predictor and the two behavioral measures entered simultaneously as dependent variables. The overall model fit was evaluated using Wilks’ lambda, which provides a multivariate test of whether hippocampal volume significantly predicts the combined outcomes. When the multivariate effect was significant, we conducted follow‐up univariate regressions to examine the contribution of hippocampal volume to each outcome separately. For each test, we report the F statistic, degrees of freedom, and *p*‐value. To estimate the magnitude of the effects, we calculated partial *η* ^2^, which represents the proportion of variance in the dependent variable uniquely explained by hippocampal volume after accounting for error variance. Following conventional benchmarks (Cohen 1988), partial *η* ^2^ values of 0.01, 0.06, and 0.14 are considered small, medium, and large, respectively.

### 2.5 Group Comparisons of Hippocampal Volume

To assess structural differences across groups, we extracted bilateral hippocampal volumes (left + right) and conducted one‐way ANOVAs. For the comparison among HY, HO, and patients with MCI, hippocampal volumes were residualized for TIV before analysis. To examine heterogeneity within the HO group, participants were additionally stratified by MoCA scores (high vs. low); in this comparison (HO‐high, HO‐low, and MCI), hippocampal volumes were residualized for both age and TIV. Significant main effects were followed by Bonferroni‐corrected post hoc tests. All tests were two‐tailed with *α* = 0.05, effect sizes are reported as *η* ^2^, and results are visualized as bar plots with individual data points.

### 2.6 Whole‐Brain Gray Matter (GM) Analyses

To complement the region‐of‐interest analyses of hippocampal volume, we performed whole‐brain voxel‐wise comparisons of GM volume. Structural MRI data were preprocessed in MATLAB (MathWorks, Natick, MA; version 2023a) using SPM12 (Wellcome Centre for Human Neuroimaging, London, UK) in combination with the CAT12 toolbox (version 12.8.2), following the VBM protocol described in (Ashburner and Friston 2000). Preprocessing included denoising with a spatially adaptive non‐local means filter, segmentation into GM, white matter (WM), and cerebrospinal fluid (CSF), and estimation of TIV (TIV = GM + WM + CSF). Segmented images were normalized to Montreal Neurological Institute (MNI) space using DARTEL at 1 mm isotropic resolution, modulated to preserve volumetric information, and smoothed with an 8 mm full‐width at half maximum (FWHM) Gaussian kernel.

Voxel‐wise analyses were conducted using SPM12 GLMs to compare patients with MCI and HO. Two‐sample, two‐tailed *t*‐tests were used, with age and TIV included as covariates. Statistical maps were thresholded at *p* < 0.05, family‐wise error (FWE) corrected, with a cluster extent of *k* = 80 voxels. Significant clusters were localized with the Automated Anatomical Labeling Atlas 3 (AAL3) (Rolls et al. 2020).

## 3 Results

### 3.1 Hippocampal Volume Predicts Memory and Bodily Awareness

**FIGURE 1:**
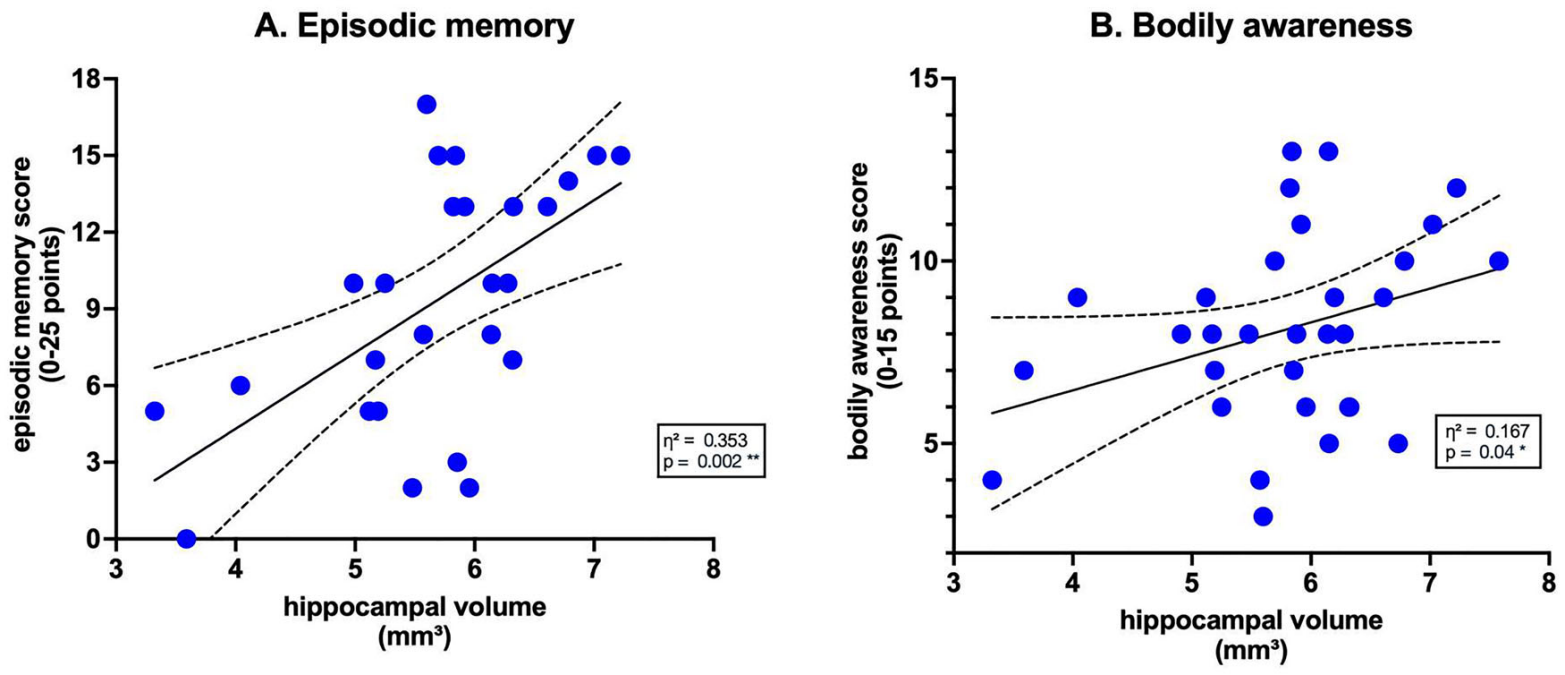
Associations between hippocampal volume and behavioral outcomes in MCI patients (*N* = 25). Scatterplots showing significant positive relationships between age‐ and TIV‐residualized hippocampal volume (*x*‐axis) and (A) episodic memory scores (delayed recall, Logical Memory Story B) and (B) bodily awareness scores (ARSQ 2.0 Somatic awareness subscale). Each point represents one MCI participant, with regression lines and 95% confidence intervals shown. Univariate follow‐up tests revealed significant associations for both memory (*F*(1, 23) = 12.56, *p* = 0.002, partial *η* ^2^ = 0.353) and bodily awareness (*F*(1, 23) = 4.60, *p* = 0.043, partial *η* ^2^ = 0.167), derived from a multivariate regression (Wilks' *Λ* = 0.61, *F*(2, 22) = 7.00, *p* = 0.004, partial *η* ^2^ = 0.389).

A multivariate regression analysis revealed that age‐ and TIV‐residualized hippocampal volume significantly predicted the combined outcomes of memory and bodily awareness in MCI patients, with Wilks’ *Λ* = 0.61, *F*(2, 22) = 7.00, *p* = 0.004, and partial *η* ^2^ = 0.389. Follow‐up univariate tests revealed that hippocampal volume was a significant predictor of memory performance, *F*(1, 23) = 12.56, *p* = 0.002, partial *η* ^2^ = 0.353 (Figure 1A), as well as bodily awareness, *F*(1, 23) = 4.60, *p* = 0.043, partial *η* ^2^ = 0.167 (Figure 1B). To further test the specificity of these associations, we ran an exploratory multivariate regression including hippocampal volume as the predictor and all 10 ARSQ 2.0 subscales simultaneously as dependent variables. The overall multivariate effect was significant (Wilks’ *Λ* = 0.235, *F*(11, 13) = 3.25, *p* = 0.012); however, univariate follow‐up tests confirmed that significant associations were confined to episodic memory and somatic awareness, with no other ARSQ 2.0 subscale reaching significance (all *F*(1, 23) < 3.43, all *p* > 0.07; see Table S2). These results confirm that the hippocampal association is specific to interoceptive bodily awareness and does not extend to other dimensions of resting‐state experience. A smaller hippocampal volume was associated with reduced memory performance and diminished bodily awareness. To test the specificity of this effect, we repeated the analysis using occipital cortex volume as the predictor. These results were not significant, Wilks’ *Λ* = 0.87, *F*(2, 22) = 1.52, *p* = 0.24, confirming the regional specificity of the hippocampal effect. All reported effects were large by these benchmarks, indicating that hippocampal volume accounts for a substantial proportion of variance in both behavioral outcomes and group differences in brain structure.

### 3.2 Hippocampal Volume Declines With Age and Cognitive Status

**FIGURE 2:**
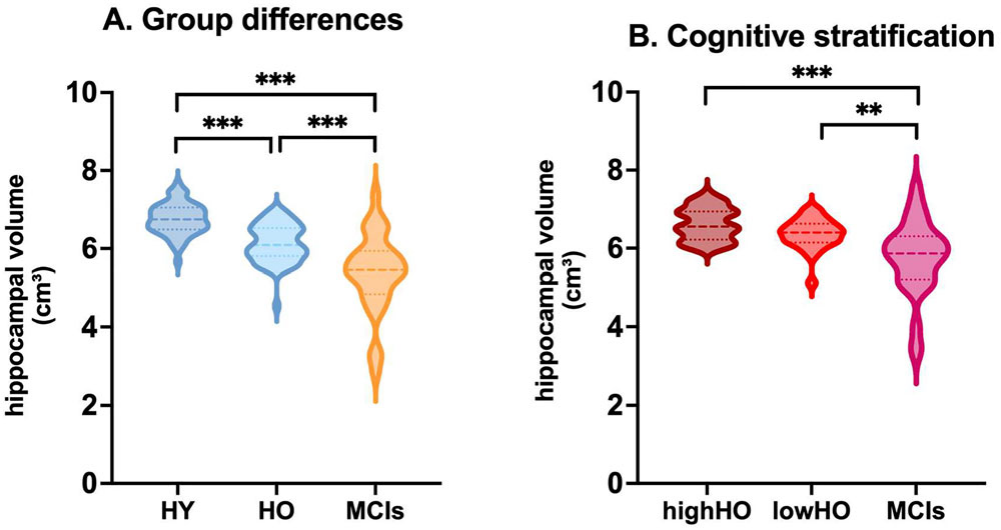
Hippocampal volume differences across groups. Violin plots with individual data points showing group differences in hippocampal volume. Within each violin, the dashed line indicates the median and the dotted lines the interquartile range (25th and 75th percentiles). (A) Hippocampal volume declined significantly across healthy young adults (HY, *N* = 58), healthy older adults (HO, *N* = 58), and patients with mild cognitive impairment (MCI, *N* = 32), with the lowest volumes observed in the MCI group. One‐way ANOVA: *F* (2, 145) =  53.01, *p* < 0.001, *η* ^2^ = 0.422. Bonferroni‐corrected post hoc tests showed that HY had significantly larger volumes than both HO (*p* < .001) and MCI (*p* < .001), and HO volumes were significantly greater than MCI (*p* < .001). (B) Stratification of HO participants by MoCA scores into high‐ and low‐performing subgroups. One‐way ANOVA: *F* (2, 87) = 13.95, *p* < 0.001, *η* ^2^ = 0.243. Bonferroni‐corrected post hoc tests showed that MCI patients had significantly lower hippocampal volumes than those in both HO‐high (*p* < 0.001) and HO‐low (*p* = 0.002) groups. The difference between HO‐high and HO‐low was not significant (exploratory). Asterisks indicate Bonferroni‐corrected post hoc significance: * *p <.01, * * *p <.001.

We examined group differences in hippocampal volume to investigate whether the relationship between hippocampal structure, memory, and bodily awareness is mediated by underlying structural integrity. Hippocampal atrophy was evaluated with a region‐of‐interest analysis comparing HY, HO, and patients with MCI. A one‐way ANOVA on TIV‐residualized hippocampal volume revealed a significant group effect, *F*(2, 145) = 53.01, *p* < 0.001, *η* ^2^ = 0.422. Post hoc comparisons showed that HY exhibited significantly larger hippocampal volumes than both HO (*p* < 0.001) and MCI patients (*p* < 0.001), and HO volumes were significantly greater than those of MCI (*p *< 0.001) (Figure 2A). This finding indicates that healthy older individuals already show diminished hippocampal volume compared to HY. To further examine the heterogeneity within the HO group, participants were stratified into high and low cognitive performers based on MoCA scores. A one‐way ANOVA on age‐ and TIV‐residualized hippocampal volume indicated significant group differences, F(2, 87) = 13.95, p < 0.001, *η* ^2^ = 0.243. Post hoc tests showed that MCI patients had significantly lower hippocampal volumes than both high‐performing HO (p < 0.001) and low‐performing HO (p = 0.002). Although the difference between high‐ and low‐performing HO participants was not significant, the low‐performing subgroup showed a tendency toward reduced hippocampal volume (Figure 2B). This exploratory pattern should be interpreted with caution; longitudinal studies with larger samples will be needed to determine whether it reflects subtle atrophy accompanying early cognitive variability within the normal range. Together, these findings demonstrate that hippocampal volume tracks age‐related decline and may also reflect cognitive variability within the healthy older population.

### 3.3 Gray Matter Loss in Hippocampal and Limbic Regions

**FIGURE 3:**
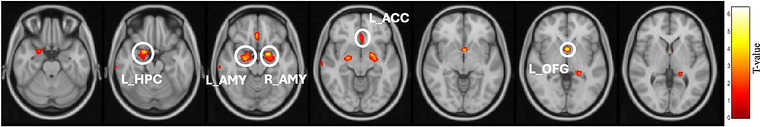
Gray matter reductions in MCI compared with healthy older adults. Axial slices showing significant clusters of reduced gray matter volume in MCI patients (*N* = 32) relative to healthy older adults (*N* = 58), based on whole‐brain voxel‐based morphometry (VBM). Two‐sample *t*‐tests with age and TIV as covariates. Statistical maps are thresholded at cluster‐level *p* < 0.05 (FWE‐corrected) with a minimum cluster size of 80 voxels. Significant reductions were observed in the left hippocampus (L_HPC**; *t* = 5.59, *p*(FWE) = 0.002**), bilateral amygdala (L_AMY**, *t* = 6.39, *p*(FWE) < 0.001**; R_AMY**, *t* = 5.80, *p*(FWE) < 0.001**), subgenual anterior cingulate cortex (L_ACC**; *t* = 6.11, *p*(FWE) < 0.001**), and left orbitofrontal gyrus (L_OFG**; *t* = 5.19, *p*(FWE) = 0.002**). The color bar indicates *t*‐values. Clusters are localized using the Automated Anatomical Labeling Atlas 3 (AAL3).

We next examined group differences in GM volume across the whole brain (cluster‐level p < 0.05, FWE‐corrected). In comparison to HO, patients with MCI exhibited pronounced reductions in medial temporal and limbic regions. Significant clusters of atrophy were detected in the bilateral amygdala (left: t = 6.39, p(FWE) < 0.001 and right: t = 5.80, p(FWE) < 0.001), the left hippocampus (t = 5.59, p(FWE) = 0.002), and the subgenual part of the anterior cingulate cortex (t = 6.11, p(FWE) < 0.001). Additional reductions were observed in the left orbitofrontal gyrus (t = 5.19, p(FWE) = 0.002) (Figure 3). These findings indicate that MCI is characterized by GM loss in a distributed network encompassing medial temporal, limbic, and prefrontal regions.

## 4 Discussion

We show that hippocampal volume predicts both episodic memory performance and bodily awareness in patients with MCI. This finding provides the first structural evidence linking the hippocampus not only to memory but also to the broader construct of BSC. By demonstrating that hippocampal atrophy is jointly associated with reduced remembering and bodily awareness, our results extend experimental and neuroimaging evidence into a clinical population with early neurodegeneration.

These results align with prior work, which shows that BSC has a critical influence on episodic encoding and retrieval (Bréchet 2022). Behavioral and neuroimaging studies have demonstrated that first‐person bodily perspectives enhance memory and that hippocampal–parietal interactions track embodiment during recollection (Bergouignan et al. 2014; Gauthier et al. 2020; Iriye and St Jacques 2021). More recently, single‐case clinical evidence revealed that hippocampal damage abolishes the mnemonic benefit of embodiment (Meyer et al. 2025). Our findings complement this by showing, at the group level, that hippocampal structural integrity predicts the degree to which memory and bodily awareness are preserved in prodromal Alzheimer's disease.

Notably, hippocampal volume accounted for substantial variance in both memory and bodily awareness, whereas occipital volume did not. This regional specificity reinforces the hippocampus as a critical locus for linking body and memory, rather than reflecting general brain atrophy. Importantly, hippocampal volume declined from that of HY to older adults and to patients with MCI. Although high‐ and low‐performing older adults did not differ significantly, the low‐performing subgroup showed a tendency toward lower volume, while both older‐adult subgroups exceeded the MCI group. This pattern is exploratory and should be interpreted cautiously; it may suggest that subtle hippocampal differences accompany cognitive variability within the healthy range but cannot be taken as evidence of preclinical decline without further longitudinal investigation.

It is worth noting that the whole‐brain VBM analysis revealed a broader pattern of GM reduction in MCI, encompassing not only the hippocampus but also the bilateral amygdala, subgenual anterior cingulate cortex, and left orbitofrontal gyrus. These regions are anatomically and functionally interconnected with the hippocampus as part of the medial temporal and limbic network, and their co‐atrophy likely reflects the distributed nature of neurodegeneration in prodromal Alzheimer's disease. The present findings should therefore be interpreted with this broader structural context in mind: while hippocampal volume emerged as a significant predictor of both memory and bodily awareness—and the occipital control analysis confirmed that this association is not simply a reflection of general brain atrophy—we cannot exclude that atrophy in connected limbic and prefrontal regions contributes to the observed behavioral deficits. Moreover, while the hippocampus emerges as a structurally significant correlate, these experiential dimensions likely arise from dynamic interactions among distributed networks that integrate mnemonic, interoceptive, and self‐related signals. The hippocampus is therefore best understood as a key node within this broader system, rather than as a structure operating in isolation (Candia‐Rivera et al. 2024). Future work may also consider the role of extra‐hippocampal regions, including the insula as a primary cortical hub for interoceptive processing, in moderating the associations reported here.

The joint association of hippocampal volume with episodic memory and bodily awareness underscores the hippocampus as a key node within the distributed network supporting autonoetic consciousness, the capacity to re‐experience past events as one's own. While hippocampal atrophy has long been recognized as a biomarker of memory decline (Mueller et al. 2010), our results suggest that its association extends to BSC. Such co‐occurring changes may contribute to the altered self‐experience and reduced interoceptive awareness reported in dementia, pointing toward novel experiential markers of early neurodegeneration.

These findings also raise translational opportunities. If replicated in longitudinal studies, systematic assessment of bodily awareness could potentially complement memory tests as a sensitive behavioral marker in aging and dementia cohorts. Moreover, interventions targeting BSC, such as virtual reality manipulations of perspective or interoceptive training (e.g., focusing on breath or heartbeat) (Ziegler et al. 2019), may mitigate memory decline by engaging hippocampal networks. Our finding that hippocampal structure relates to bodily awareness dovetails with work showing that interoceptive rhythms influence awareness‐related processing (Leupin and Britz 2024), suggesting that training aimed at interoception and embodiment could leverage both perceptual and mnemonic pathways. In particular, meditation‐based practices designed to cultivate interoceptive awareness represent a promising avenue, as they directly target bodily self‐experience, have been established as feasible in older adults, and could be leveraged to slow or buffer hippocampal‐related decline in at‐risk populations. Likewise, non‐invasive brain stimulation techniques, including transcranial alternating current stimulation (Koch et al. 2024; Benussi et al. 2022), show potential to improve hippocampal network function and memory, and could be extended to embodied self‐processes. Together, these approaches highlight that targeting the embodied self may provide novel routes to maintain memory and selfhood in aging and disease.

### 4.1 Limitations and Future Directions

Bodily awareness in this study was operationalized as interoceptive bodily awareness, the dimension of resting‐state experience most directly relevant to BSC and the only one in the ARSQ 2.0 explicitly capturing somatic self‐reference. While this choice is hypothesis‐driven and empirically supported by the specificity analysis, interoceptive awareness represents one facet of a broader construct that also encompasses proprioception, vestibular signals, body ownership, and first‐person perspective‐taking. Future work should employ more comprehensive measures, including experimental manipulations of embodiment, to determine whether hippocampal associations with BSC extend beyond the interoceptive domain.

Furthermore, the regression analyses were conducted in a relatively small sample of MCI patients (*n* = 25), and effect size estimates derived from small samples are known to be inflated; the large partial *η* ^2^ values reported here should therefore be interpreted with caution and await replication in larger samples.

It should be noted that the sex distribution differed across groups, with fewer female participants in the MCI group (41%) relative to healthy young (55%) and older adults (57%), and sex was not included as a covariate. Future studies should examine whether sex moderates the reported associations.

A further concern is that the ARSQ relies on retrospective self‐report, which may itself be compromised in individuals with hippocampal atrophy, potentially introducing a circularity in the interpretation of our findings. Future studies should consider online or experience‐sampling measures of bodily awareness.

Finally, longitudinal studies are needed to determine whether reduced bodily awareness predicts conversion from MCI to Alzheimer's disease, thereby potentially serving as an early clinical marker of progression—a question that remains to be tested.

## 5 Conclusion

In sum, our study identifies hippocampal atrophy as a structural correlate of both reduced memory performance and diminished bodily awareness in MCI. While the hippocampus emerges as a significant node in this association, our findings are best understood within the broader distributed network—encompassing medial temporal, limbic, and prefrontal regions—that integrates mnemonic, interoceptive, and self‐related processes. Together, these results provide a promising foundation for future research aimed at early detection and intervention in neurodegeneration.

## Author Contributions

**Paul G. Unschuld**: writing – review and editing. **Lucie Bréchet**: writing – original draft, funding acquisition, methodology, supervision, conceptualization, investigation, resources, formal analysis. **Damien Marie**: writing – review and editing, validation, methodology, formal analysis, supervision. **Konstantin Toussas**: investigation, writing – review and editing, methodology, formal analysis.

## Funding

The Dementia Research Switzerland– Synapsis Foundation (Grant No. 2024‐CAD03) and Swiss National Science Foundation (Grant No. PZ00P1_208686) supported this work.

## Conflicts of Interest

The authors declare no conflicts of interest.

## Supporting information




**Supplementary Materials**: brb371570‐sup‐0001‐Tables.docx

## Data Availability

The datasets analyzed during the current study are available from the corresponding author upon reasonable request. The included demographic, neuropsychological, and neuroimaging data are fully anonymized, and no patient or subject's personal and sensitive information is at risk.
