# Supplementary material for: The Hippocampus Links Episodic Memory and Bodily Awareness
Source: Brain Behav. 2026 Jul 8;16(7):e71570. doi: 10.1002/brb3.71570 (PMC13344893; doi:10.1002/brb3.71570)
Supplement: Supplementary file 1 — Supplementary Materials: brb371570‐sup‐0001‐Tables.docx [file BRB3-16-e71570-s001.docx]

**Supplementary Material**

**SUPPLEMENTARY TABLE S1**

| **Dimension** | **Items** |
| --- | --- |
| **Somatic Awareness*** | ***“I was conscious of my body.”*** |
|  | ***“I thought about my heartbeat.”*** |
|  | ***“I thought about my breathing.”*** |
| **Self** | *“I thought about my feelings.”* |
|  | *“I thought about my behaviour.”* |
|  | *“I thought about myself.”* |
| **Sleepiness** | *“I felt tired.”* |
|  | *“I felt sleepy.”* |
|  | *“I had difficulty staying awake.”* |
| **Comfort** | *“I felt comfortable.”* |
|  | *“I felt relaxed.”* |
|  | *“I felt happy.”* |
| **Health Concern** | *“I felt ill.”* |
|  | *“I thought about my health.”* |
|  | *“I felt pain.”* |
| **Discontinuity of Mind** | *“I had busy thoughts.”* |
|  | *“I had rapidly switching thoughts.”* |
|  | *“I had difficulty holding on to my thoughts.”* |
| **Theory of Mind** | *“I thought about others.”* |
|  | *“I thought about people I like.”* |
|  | *“I placed myself in other people's shoes.”* |
| **Planning** | *“I thought about things I have to do.”* |
|  | *“I thought about solving problems.”* |
|  | *“I thought about the future.”* |
| **Visual Thoughts** | *“I thought in images.”* |
|  | *“I pictured events.”* |
|  | *“I pictured places.”* |
| **Verbal Thoughts** | *“I thought in words.”* |
|  | *“I had silent conversations.”* |
|  | *“I imagined talking to myself.”* |

**Supplementary Table S1.** Amsterdam Resting-State Questionnaire (ARSQ 2.0) — dimensions and items. The questionnaire comprises 30 items across 10 dimensions, each rated on a 5-point Likert scale (1 = completely disagree, 5 = completely agree). The Somatic Awareness subscale (highlighted) was selected a priori as the only dimension directly capturing interoceptive and bodily self-referential experience.

** Subscale used in the present study. Items are rated on a 5-point Likert scale (1 = completely disagree, 5 = completely agree).*

**SUPPLEMENTARY TABLE S2**

| **ARSQ 2.0 Subscale / Outcome** | **F(1, 23)** | **p** | **Partial η²** |
| --- | --- | --- | --- |
| **Episodic memory** | **12.56** | **.002 *** | **.353** |
| **Somatic Awareness** | **4.61** | **.043 *** | **.167** |
| Self | 1.69 | .207 | .068 |
| Sleepiness | 2.20 | .151 | .087 |
| Comfort | 3.12 | .091 | .119 |
| Health Concern | 3.42 | .077 | .130 |
| Discontinuity of Mind | 1.86 | .186 | .075 |
| Theory of Mind | 0.13 | .723 | .006 |
| Planning | 0.06 | .808 | .003 |
| Visual Thought | 0.61 | .438 | .026 |
| Verbal Thought | 0.33 | .574 | .014 |

**Supplementary Table S2.** *Univariate follow-up tests from multivariate regression of hippocampal volume on all ARSQ 2.0 subscales and episodic memory.* *F-values reflect the hippocampal volume effect in a multivariate GLM with age- and TIV-residualized hippocampal volume as covariate. Overall multivariate test: Wilks' Λ = .235, F(11, 13) = 3.25, p = .012. * p < .05. Shaded rows indicate significant effects.*
